# The Glucocorticoid PYED-1 Disrupts Mature Biofilms of *Candida* spp. and Inhibits Hyphal Development in *Candida albicans*

**DOI:** 10.3390/antibiotics10111396

**Published:** 2021-11-13

**Authors:** Anna Esposito, Antonella Migliaccio, Vita Dora Iula, Raffaele Zarrilli, Annalisa Guaragna, Eliana De Gregorio

**Affiliations:** 1 Department of Chemical, Materials and Production Engineering, University of Naples Federico II, 80126 Naples, Italy; anna.esposito5@unina.it (A.E.); annalisa.guaragna@unina.it (A.G.); 2 Department of Public Health, University of Naples Federico II, 80131 Naples, Italy; antonella.migliaccio10@gmail.com (A.M.); rafzarri@unina.it (R.Z.); 3 Complex Operative Unit of Clinical Pathology, Ospedale del Mare-ASL NA1 Centro, 80145 Naples, Italy; dora.iula@gmail.com; 4 Department of Molecular Medicine and Medical Biotechnology, University of Naples Federico II, 80131 Naples, Italy

**Keywords:** glucocorticoid, antifungal agents, anti-biofilm agents, *Candida* spp.

## Abstract

Invasive *Candida* infections have become a global public health problem due to the increase of *Candida* species resistant against antifungal therapeutics. The glucocorticoid PYED-1 (pregnadiene-11-hydroxy-16α,17α-epoxy-3,20-dione-1) has antimicrobial activity against various bacterial taxa. Consequently, it might be considered for the treatment of *Candida* infections. The antifungal activity of PYED-1 was evaluated against several fungal strains that were representative of the five species that causes the majority of *Candida* infections—namely, *Candida albicans*, *Candida glabrata*, *Candida tropicalis*, *Candida parapsilosis* and *Candida krusei*. PYED-1 exhibited a weak antifungal activity and a fungistatic effect on all five *Candida* species. On the other hand, PYED-1 exhibited a good anti-biofilm activity, and was able to eradicate the preformed biofilms of all *Candida* species analyzed. Moreover, PYED-1 inhibited germ tube and hyphae formation of *C. albicans* and reduced adhesion of *C. albicans* to abiotic surfaces by up to 30%.

## 1. Introduction

Candidemia and invasive candidiasis are severe healthcare-associated infections with a high mortality rate and limited therapeutic options [1,2]. *Candida* *albicans*, *Candida* *glabrata*, *Candida* *tropicalis*, *Candida* *parapsilosis* and *Candida* *krusei* are the five species that cause approximately 90% of all infections [3]. *C. albicans* is the most prevalent cause of invasive fungal infections. The therapeutic failures of *C. albicans* infections are quite high [4], although the levels of antifungal resistance reported for this organism are very low [5]. Recent studies reported a growing proportion of non-*albicans Candida* infections [6]. Patients with a non-*albicans Candida* infection have a less effective response to antifungal therapies and, as a consequence, a high degree of mortality [4]. *Candida* spp. can form biofilm on biotic and abiotic surfaces, becoming less susceptible to antifungal drugs. Biofilm formation is highly associated with persistent candidemia [7].

The increasing resistance of fungal species to antifungal drugs has led to a need for the development of novel therapeutic agents with a broad spectrum of activity, along with low toxicity and high efficacy against all *Candida* species.

**Figure 1:**
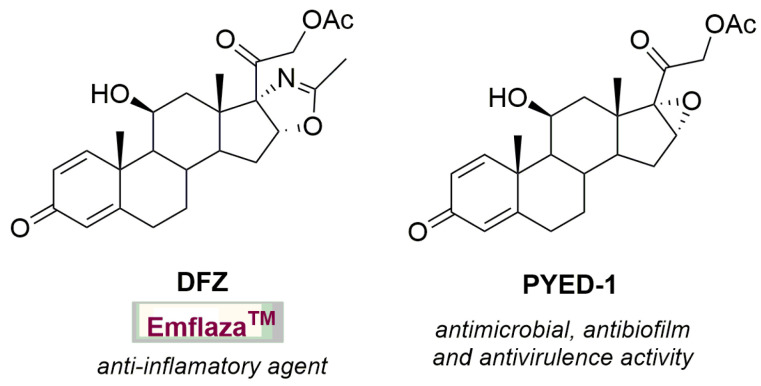
The corticosteroid drug deflazacort (DFZ) and its synthetic precursor PYED-1.

As part of our interest in the synthesis of compounds endowed with pharmacological activity [8,9,10,11,12], we recently developed a novel synthetic route aimed at the preparation of the corticosteroid drug deflazacort (DFZ, Figure 1) in order to explore its antimicrobial activity. 

In this context, while DFZ showed no activity, one of its synthetic precursors, the heterocyclic corticosteroid PYED-1 (pregnadiene-11-hydroxy-16α,17α-epoxy-3,20-dione-1), showed significant antimicrobial, antibiofilm and anti-virulence activities against Gram-positive and Gram-negative bacteria without showing cytotoxicity at concentrations up to 128 µg/mL [13,14,15,16]; nevertheless, its ability to interfere with fungal pathogens has not yet been investigated. Herein we describe the evaluation of the corticosteroid PYED-1 as an inhibitor of the growth and biofilm formation in five species of *Candida*, namely, *C. albicans*, *C. glabrata*, *C. tropicalis*, *C. parapsilosis* and *C. krusei*.

## 2. Results and Discussion

### 2.1. Antifungal Susceptibility to Glucocorticoid PYED-1 of Candida Species

The minimal inhibitory concentration (MIC) and minimal lethal concentration (MLC) of PYED-1 were determined for *C. albicans*, *C. glabrata*, *C. parapsilosis*, *C. tropicalis* and *C. krusei* clinical isolates. The drug fluconazole was used as a positive control. Results in Table 1 show that PYED-1 had anticandidal activity on all clinical isolates of all *Candida* spp. assayed.

PYED-1 exhibited weak inhibitory activity against all *Candida* species with MIC values ranging from 16 μg/mL to 128 μg/mL, and the effect was fungistatic for the MIC values.

To test whether PYED-1 can synergize with available antifungal drugs, checkerboard assays were performed. Fluconazole or posaconazole had neither synergistic nor antagonistic interactions with PYED-1 (data not shown), indicating that PYED-1 co-treatment cannot impair or attenuate the efficacy of these antifungal drugs.

### 2.2. Effect of PYED-1 on Preformed Candida *spp.* Biofilm

**Figure 2:**
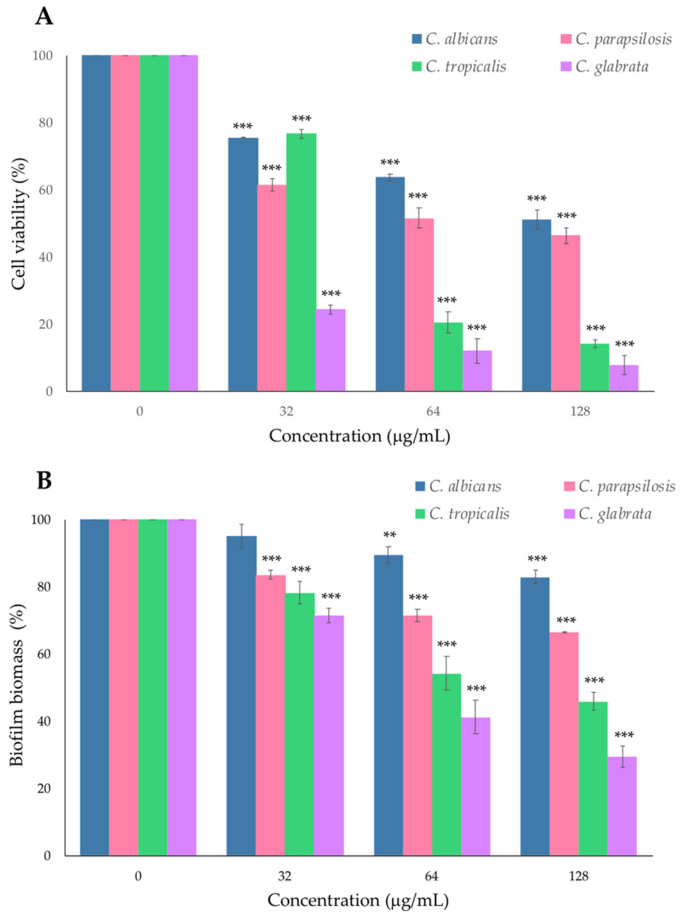
Eradication biofilm assay. The effect of treatment with 128, 64 and 32 μg/mL of PYED-1 on preformed biofilm of *Candida* species. The metabolic activities of biofilm cells (**A**) and biofilm biomass (**B**) were measured by XTT reduction assay and CV staining, respectively. All experiments were performed in triplicate. Asterisks indicate a statistically significant difference (** *p*-value ≤ 0.01 and *** *p*-value ≤ 0.001 from the nontreated controls without PYED-1).

The formation of fungal biofilm on biotic and abiotic surfaces is a major virulence factor in *Candida* spp. It causes a reduction in the penetration and, therefore, in the efficacy of antifungal drugs. It contributes to the appearance of drug resistance and recurrent fungal infections in the clinic [17]. Therefore, it is of interest to identify novel molecules with antibiofilm activity. The antibiofilm activity of PYED-1 was evaluated against preformed biofilms of *C. albicans*, *C. glabrata*, *C. parapsilosis* and *C. tropicalis* strains by XTT (2,3-bis (2-methyloxy-4-nitro-5-sulfophenyl)-2H-tetrazolium-5-carboxanilide) reduction and crystal violet (CV) assays. As shown in Figure 2, results varied according to the *Candida* species.

In the eradication biofilm assay, exposure of preformed *C. glabrata* biofilms to 128, 64 and 32 μg/mL of PYED-1 reduced the cell viability by 75–92% (Figure 2A) and the biofilm biomass by 30–70% (Figure 2B), as compared to untreated controls. PYED-1 at 64 μg/mL was able to reduce the number of viable *C. tropicalis* to over 80% (Figure 2A), while biofilm biomass was diminished by 50% (Figure 2B). In contrast, PYED-1 displayed a significant reduction in the number of viable cells of preformed biofilms of *C. albicans* and *C. parapsilosis* when compared to untreated controls, but only marginally reduced the biofilm biomass (Figure 2). *Candida* biofilms are widely considered to be a key determinant of the high mortality rate attributed to candidiasis [7]. *Candida* can form biofilms on biomedical implants that are difficult to eradicate by clinical drugs such as amphotericin B, fluconazole, flucytosine and itraconazole. They are also responsible for the high resistance to antifungal drugs [18]. Antifungal resistance due to biofilm formation is frequently reported in clinical settings, and it counteracts the achievement of effective treatment strategies [19]. Antibiofilm agents represent a novel approach to reducing antifungal resistance, destroying preformed biofilm and making the persistent cells susceptible to antifungal drugs [20]. Thus, the ability to disrupt preformed biofilms of all four *Candida* species analyzed by the death of fungal cells wrapped by the extracellular matrix suggests a potential therapeutic role for PYED-1.

### 2.3. PYED-1 Inhibited the Filamentation and Adhesion of C. albicans

**Figure 3:**
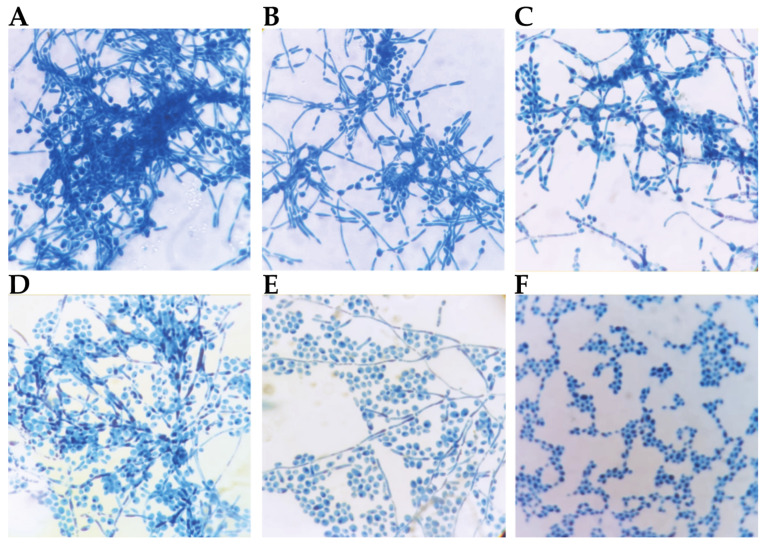
Light-microscopy images taken at 100× magnification of *C. albicans* ATCC 10231 stained with 0.3% crystal violet, without (**A**) or with the addition of PYED-1 solutions at 2 µg/mL (**B**), 4 µg/mL (**C**), 8 µg/mL (**D**), 16 µg/mL (**E**) and 32 µg/mL (**F**).

Mature *Candida* biofilms are formed by a mixture of yeast cells, pseudohyphae and hyphae [21], and the dimorphic switch of yeast cells to hyphal cells is required for the maturation of structured biofilms by *C. albicans* [22]. The transition of yeast cells to hyphae is considered an important virulence factor of *C. albicans*: it can promote fungal invasion of human mucosal tissues, cause invasive fungal infections and aid in escape from immune cells [23]. Hyphal formation is associated with virulence, as genes encoding virulence factors are coregulated with hypha-specific genes [23]. The ability of PYED-1 to inhibit *C. albicans* filamentation at different concentrations after 5 h of incubation at 37 °C was investigated by microscopy (Figure 3).

The filamentation of *C. albicans* induced by serum was effectively suppressed in the presence of PYED-1 in RPMI-FBS (fetal bovine serum) medium in a concentration-dependent manner. After 5 h of drug exposure, PYED-1 fully inhibited hyphae formation of *C. albicans* ATCC 10231 strain at 32 μg/mL, as compared to the untreated control. Microscopic images of *C. albicans* untreated or treated with 2 μg/mL and 4 μg/mL of PYED-1 formed long hyphae (Figure 3A–C). In cells treated with 8 μg/mL and 16 μg/mL of the drug, fewer filaments and more yeasts per field were observed (Figure 3D,E). Microscopic images of *C. albicans* cells treated with 32 μg/mL of PYED-1 showed yeast cells but no filaments (Figure 3F). Many molecules induce changes in cell wall components associated with the transition from yeast to hyphal growth and/or reduce the expression of various virulence factors [24]. Molecules with antimicrobial activity, which exhibit a synergistic effect with conventional drugs or reduce the expression of pathogen virulence factors, can represent new treatment options that are effective in combating fungal infections [24,25]. The prevention of hyphae growth by PYED-1 is a significant result since yeast-to-hypha transition is a critical virulence feature which mediates *C. albicans* invasion into infected tissue.

**Figure 4:**
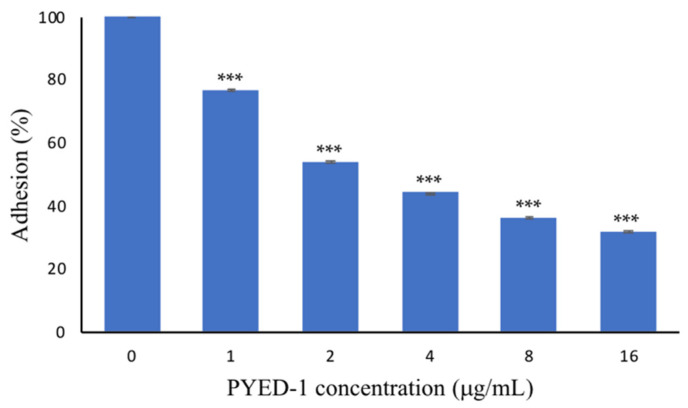
The adhesion of *C. albicans* with the addition of different concentrations of PYED-1. The adhesion was measured after 90 min of addition of PYED-1 by CV staining. All experiments were performed in triplicate. Asterisks indicate a statistically significant difference (*** *p*-value ≤ 0.001 from the nontreated controls without PYED-1).

A hyperfilamentous strain of *C. albicans* is characterized by a high adhesion ability [26]. Hypha-specific genes encode proteins that are involved in the adhesion to host cells [23]. Strong adherence to cell/tissue or the surfaces of medical devices is the earliest stage of a *Candida* infection. Since PYED-1 influenced the yeast-to-hyphal morphological transition, we thought it interesting to check whether this compound could affect cell adhesion to abiotic surfaces. As shown in Figure 4, PYED-1 significantly reduced the adhesion of *C. albicans* ATCC 10,231 to polystyrene surface by up to 30% compared with the negative control.

PYED-1 exhibited a 46% and 55% reduction in the adhesion of *C. albicans* at sub-MIC concentrations of 2 µg/mL and 4 µg/mL, respectively. At the highest concentration used (16 µg/mL), PYED-1 decreased about 70% of the adhesion, as compared to the untreated controls. PYED-1 can inhibit the adherence of *C. albicans* and the formation of hyphae. This suggests that PYED-1 may prevent the colonization and tissue penetration of *C. albicans*. Several studies have shown that adherence and hyphae formation are closely connected. The *ALS* gene family, encoding a set of cell glycoproteins that promote fungal adherence and biofilm formation, is expressed specifically during hyphal development [27]. Hwp1, a *C. albicans* cell-surface protein expressed only on hyphae, plays an important role in hyphal development, but is also required for fungal adherence and biofilm formation [27,28].

Blastospores, pseudohyphae and hyphae play a critical role in the process of *C. albicans* biofilm development and maturation, providing stability to the biofilm structure [23]. Therefore, inhibition of the adhesion of yeast cells and their differentiation in filamentous form can represent an effective therapeutic alternative [29]. In light of this, the glucocorticoid PYED-1 with a broad antimicrobial activity has the potential to serve as a preventive tool. It functions by affecting the adhesion of yeast cells and their differentiation in filamentous form, and by inhibiting existing or forming *C. albicans* biofilms. However, the specific antibiofilm mechanism of PYED-1 has not yet been described. Future studies will be necessary to understand the mode of action of PYED-1 and to evaluate its therapeutic use during *Candida* spp. infections.

## 3. Materials and Methods

### 3.1. Fungal Strain, Culture Condition and Compound Synthesis

*Candida* strains evaluated in this study included the *C. albicans* ATCC 10231 reference strain and 6–8 clinical isolates of each *Candida* species belonging to a bacterial collection that was previously established. No ethical approval was required for this study as there was no access to patients’ data. Strains were routinely grown on Sabouraud dextrose agar (SDA) and in RPMI-1640 broth buffered with MOPS (pH 7.0) containing glucose 2% (RPMI-1640 2% G) at 37 °C. The chemical synthesis and structural characterization of PYED-1 were accomplished as previously reported [13]. Stock solutions of the compounds at the concentration of 50 mg/mL were prepared in dimethyl sulfoxide (DMSO) and kept at −20 °C and diluted in RPMI-1640 2% G broth just prior to the assays.

### 3.2. Determination of the Minimum Inhibitory Concentration (MIC) and the Minimum Fungicide Concentration (MFC)

PYED-1 was evaluated for its in vitro antifungal activities according to EUCAST guidelines using the two-fold serial dilution technique in 96-well microtiter plates [30]. Briefly, yeasts from overnight cultures on SDA were suspended in RPMI-1640 2% G broth to get 1–5 × 10^5^ colony-forming units (cfu)/mL. Two-fold serial dilutions of PYED-1 were prepared in RPMI-1640 2% G medium, starting from 512 to 1 µg/mL, in order to obtain a 2× final concentration of the antifungal agent. Each well of plate was inoculated with 100 µL of 1–5 × 10^5^ cfu/mL yeast suspension and 100 µL of twice the final antifungal agent PYED-1 or 100 µL of sterile drug-free medium (growth control). A sterility control containing 200 mL RPMI-1640 medium was included in each plate. The plate was incubated without agitation at 37 °C for 24–28 h. The optical density at 450 nm was measured using a microplate reader (Bio-Rad Laboratories S.r.l., Segrate (MI) - Italy). MIC was the lowest concentration inhibiting ≥90% growth when compared to growth control. The effect of different concentrations of DMSO (ranging from 0.1% to 0.5%) on fungal growth was separately tested. In order to evaluate the minimum lethal concentration (MLC, i.e., the lowest concentration showing a growth inhibition of 100%), 20 µL of yeast suspensions from wells without visible growth were transferred to SDA plates. These plates were incubated at 37 °C and checked for growth after 24 h. All experiments were performed in triplicate and were repeated independently at least three times.

### 3.3. Checkboard Microdilution Assay

The checkerboard method was used to evaluate the MICs for PYED-1 alone and in combination with the antifungal agents fluconazole and posaconazole for *C. parapsilosis* 84,609 and 84,614, *C. glabrata* 61,115 and *C. albicans* 61,446 and 61,691 strains, as described previously [31]. The plates were incubated at 37 °C for 24–28 h. The fractional inhibitory concentration index was calculated for the compound combinations, with a FICI < 0.5 indicating “synergism”, a FICI > 4 indicating “antagonism”, and a FICI between 0.5 and 4 defined as “without interaction” [32]. The results were obtained from tests that were repeated three times.

### 3.4. Germ Tube Inhibition Assay

To determine serum-induced cell filamentation, *C. albicans* ATCC 10,231 cells were inoculated in 2 mL of RPMI-1640 medium containing 10% (*v*/*v*) FBS at a density of 10^6^ cfu/mL in a glass tube without (control) or with the addition of PYED-1 solutions at 2, 4, 8, 16 and 32 µg/mL. After a 5 h incubation at 37 °C, 30 µL cells from treated and untreated samples were smeared, fixed, stained with 0.3% crystal violet and observed under a light microscope.

### 3.5. Adhesion Assay

The adhesion prevention efficacy of PYED-1 was assayed using the crystal violet biofilm staining method [33]. Cultures of *C. albicans* ATCC 10,231 were grown in RPMI-1640 2% G and incubated overnight at 30 °C with agitation (100 rpm). Cells were harvested and washed with PBS and diluted to a density of 1 × 10^6^ cfu/mL in RPMI-1640 2% G. The wells of polystyrene flat-bottom 96-well microtiter plates were conditioned with 50% FBS in PBS for at least 30 min at room temperature. Then, the FBS was aspirated and wells were rinsed once with PBS. One hundred microliters of the yeast suspension was added to each well containing 100 µL of twice the final concentration of antifungal agent PYED-1 (2, 4, 8, 16 and 32 µg/mL) or 100 µL of sterile drug-free medium (growth control). The plate was incubated at 37 °C for 90 min. Then, the medium was aspirated, wells were washed with PBS twice and 100 µL of 0.1% crystal violet was added to each well. After 15 min, the plate was washed two times and CV was dissolved with 200 µL of 100% ethanol. Absorbance was measured at 595 nm using a microplate reader (Bio-Rad Laboratories S.r.l.). The percentage of biofilm mass reduction was calculated as [(Ac−At)/Ac] × 100, where Ac is the OD_595_ for the control well and At is the OD_595_ for the biofilm in the presence of PYED-1. All data points are expressed as means + SDs of three separate experiments performed in triplicate.

### 3.6. Anti-Preformed-Biofilm Assay

Biofilms were allowed to form in each well as described in the adhesion assay and were treated following 24 h of incubation. After the adhesion step, nonadherent cells were removed and wells were washed with PBS. Two hundred microliters of RPMI-1640 2% G was added to each well and subsequently incubated at 37 °C for 18–24 h in an orbital shaker (100 rpm). After incubation, wells were washed with PBS and 200 µL of twice the final antifungal agent PYED-1 (64, 128 and 256 µg/mL) or 100 µL of sterile drug-free medium (growth control) was added. Biofilm was incubated statically at 37 °C for an additional 18–24 h. After PYED-1 treatment, the metabolic activities of biofilm cells and biofilm biomass were measured by XTT reduction assay and CV staining [34], respectively. Briefly, 200 μL of a solution of XTT (0.5 mg/mL in PBS; Sigma, Milan, Italy) menadione (1 µM in acetone; Sigma) was added to each prewashed well. Following incubation in the dark for up to 2 h at 37 °C, the biofilm metabolic activity was determined through the measurement of the absorbance value at 490 nm using a microplate reader (Bio-Rad Laboratories S.r.l.). Viability values were compared with respect to control samples treated with scalar doses of DMSO (concentrations ranging from 0.064% to 0.256%). All data points are expressed as means + SDs of three separate experiments performed in triplicate.

### 3.7. Statistical Analysis

All statistical analyses were performed with GraphPad Prism 8 software (GraphPad, San Diego, CA, USA). All results are presented as arithmetic means ± standard deviations. Statistical differences between PYED-1 treated and untreated samples were analyzed by one-way analysis of variance (ANOVA) followed by Student’s *t*-test. Statistical significance was considered at *p*-value of <0.05.

## Figures and Tables

**Table 1:** Minimal inhibitory concentration (MIC) and minimal lethal concentration (MLC) of PYED-1 against *Candida* species.

Species	Strain	PYED-1		Fluconazole
		MIC (µg/mL)	MLC (µg/mL)	MIC (g/mL)
*C. albicans*	ATCC 10231	16	64	1
	61142	16	64	0.5
	61280	16	64	0.5
	61446	32	64	128
	61540	16	64	0.5
	61558	32	128	1
	61691	32	64	64
	62020	16	64	0.5
	62033	16	64	1
*C. glabrata*	60932	32	64	0.5
	60940	32	64	0.5
	61112	16	128	1
	61115	32	128	4
	65821	64	>128	1
	67676	64	>128	1
	81263	32	64	0.5
*C. parapsilosis*	60568	32	128	0.5
	61446	64	>128	2
	66627	128	>128	0.5
	80149	128	>128	1
	81208	64	>128	0.5
	81879	128	>128	0.5
	84609	128	>128	32
	84614	128	>128	32
*C. tropicalis*	60981	16	32	0.5
	61220	64	>128	1
	81222	32	64	0.5
	81252	32	64	0.5
	81419	64	>128	0.5
	82193	32	128	1
*C. krusei*	61159	32	>128	32
	67053	32	>128	64
	69788	32	>128	32
	71456	32	>128	32
	81288	32	128	16
	81667	32	>128	32
